# The mammalian cytosolic thioredoxin reductase pathway acts via a membrane protein to reduce ER-localised proteins

**DOI:** 10.1242/jcs.241976

**Published:** 2020-04-30

**Authors:** Xiaofei Cao, Sergio Lilla, Zhenbo Cao, Marie Anne Pringle, Ojore B. V. Oka, Philip J. Robinson, Tomasz Szmaja, Marcel van Lith, Sara Zanivan, Neil J. Bulleid

**Affiliations:** 1 Institute of Molecular, Cell and Systems Biology, College of Medical Veterinary and Life Sciences, Davidson Building, University of Glasgow, Glasgow, G12 8QQ, UK; 2 Cancer Research UK Beatson Institute, Glasgow, G61 1BD, UK; 3 Institute of Cancer Sciences, University of Glasgow, Glasgow, G61 1QH, UK

**Keywords:** Disulfide formation, Endoplasmic reticulum, Protein folding, Thioredoxin pathway

## Abstract

Folding of proteins entering the mammalian secretory pathway requires the insertion of the correct disulfides. Disulfide formation involves both an oxidative pathway for their insertion and a reductive pathway to remove incorrectly formed disulfides. Reduction of these disulfides is crucial for correct folding and degradation of misfolded proteins. Previously, we showed that the reductive pathway is driven by NADPH generated in the cytosol. Here, by reconstituting the pathway using purified proteins and ER microsomal membranes, we demonstrate that the thioredoxin reductase system provides the minimal cytosolic components required for reducing proteins within the ER lumen. In particular, saturation of the pathway and its protease sensitivity demonstrates the requirement for a membrane protein to shuttle electrons from the cytosol to the ER. These results provide compelling evidence for the crucial role of the cytosol in regulating ER redox homeostasis, ensuring correct protein folding and facilitating the degradation of misfolded ER proteins.

## Abstract (precis)

**Summary:** Correct disulfuide formation in the secretory pathway requires reducing equivalents from the cytosol mediated through a membrane protein localised to the endoplasmic reticulum.

## INTRODUCTION

Proteins entering the secretory pathway undergo several modifications that are unique to the ER, including glycosylation and disulfide formation (Braakman and Bulleid, 2011). The consequence of these modifications is often increased stability of the protein fold in preparation for secretion into the extracellular milieu. Failure to fulfil the correct modification can lead to protein misfolding and disease (Wang and Kaufman, 2016). Correct protein disulfide formation requires resident ER proteins to catalyse both disulfide formation and reduction (Ellgaard et al., 2018). Disulfide formation is catalysed primarily by Ero1, which oxidises members of the protein disulfide isomerase (PDI) family by coupling the reduction of oxygen to the introduction of a disulfide (Sevier et al., 2007). PDI then exchanges its disulfide with substrate proteins during and following their translocation into the ER lumen (Chen et al., 1995). During this process, disulfides might form that are not present in the final native structure (Jansens et al., 2002). Such non-native disulfides need to be removed to allow correct folding or to facilitate protein degradation (Ushioda et al., 2008). Hence, there is a requirement for both an oxidative and reductive pathway in the ER to ensure that correct native disulfides are formed or for proteins to be targeted for degradation (Bulleid and Ellgaard, 2011). In addition to the reduction of structural disulfides, there is also a requirement for a reductive pathway to recycle enzymes such as methionine sulfoxide reductase (Cao et al., 2018), vitamin K epoxide reductase (Rishavy et al., 2011), peroxiredoxin IV (Tavender et al., 2010) and formyl glycine generating enzyme (Dierks et al., 2005), which contain active site thiols that become oxidised during catalysis.

Despite our understanding of the various pathways to introduce disulfides, our knowledge of the reductive pathway is limited. Members of the PDI family such as ERdj5 are likely to catalyse the initial stage in the reduction of non-native disulfides (Jessop et al., 2007; Oka et al., 2013; Ushioda et al., 2008). ERdj5 has a relatively low reduction potential, making it an efficient reductase (Hagiwara et al., 2011). ERdj5 is recruited to substrate proteins via its interaction with BiP (Cunnea et al., 2003; Hagiwara et al., 2011; Oliver et al., 1997). Following the reduction of substrates, the PDI reductases become oxidised so there is a requirement for their enzymatic reduction to maintain activity. Within the cytosol, the main disulfide reductase, thioredoxin-1 (Trx1), is maintained in a reduced state by the action of cytosolic thioredoxin reductase 1 (TrxR1) with potential contributions from both the glutathione reductase (GR) and glutaredoxin (Grx) pathways (Lillig and Holmgren, 2007). Both the Trx1 and glutathione (GSH) pathways require NADPH as ultimate electron donor to maintain their reductase activity within the cytosol. However, there are no known equivalent pathways present within the ER for the reduction of disulfides in the thioredoxin domains within the PDI family. We recently showed that the regeneration of NADPH within the cytosol is required to ensure correct disulfide formation in the ER lumen (Poet et al., 2017), raising the possibility that the cytosolic reductive pathways are responsible for ensuring correct disulfide formation in the ER lumen. How the reducing equivalents generated in the cytosol are transferred to the ER lumen remains unknown, but a system for the transfer of such equivalents does exist in prokaryotes. There, disulfide formation and reduction within the periplasmic space is catalysed by DsbA and DsbC, which are structurally homologous to Trx1 (Kadokura et al., 2003). Disulfide formation is coupled to the electron transport chain via a membrane protein called DsbB, allowing *de novo* disulfide formation in the disulfide exchange protein DsbA. To remove incorrectly oxidised periplasmic thiols, the cytosolic thioredoxin reductase pathway via thioredoxin reduces the membrane protein DsbD, which transfers electrons across the membrane to reduce DsbC that then catalyses disulfide reduction.

A role for GSH in the reduction of protein thiols has been suggested based on its role as a redox buffer (Chakravarthi et al., 2006). This role is thought to be required to maintain redox balance after large fluctuations in either reducing or oxidising conditions (Appenzeller-Herzog et al., 2010; Jessop and Bulleid, 2004; Molteni et al., 2004). A recent report identifying Sec61 as a GSH transporter provides a possible route for its transfer into the ER (Ponsero et al., 2017). However, the GSH requirement for the formation of the correct disulfides in proteins is less clear. Depletion of ER GSH either by inhibition of GSH synthase or by targeting GSH-degrading enzymes does not prevent correct disulfide formation in proteins containing complex disulfides, such as tissue-type plasminogen activator or the low-density lipoprotein receptor (Chakravarthi and Bulleid, 2004; Tsunoda et al., 2014). The relative roles of the thioredoxin and GSH pathways in maintaining ER redox poise and in reducing oxidised thiols remain undefined (Bulleid and Ellgaard, 2011; Ellgaard et al., 2018).

To evaluate the requirement for the reduction of disulfides within the ER, we reconstituted the pathway using purified cytosolic components and microsomal vesicles or semi-permeabilised (SP) cells as a source of ER. Using a redox-sensitive green fluorescent protein (roGFP) as a readout (van Lith et al., 2011), we established the minimum requirements for disulfide reduction and demonstrated that the transfer of reducing equivalents across the ER membrane requires a membrane protein. In addition, we showed that the resolution of non-native to native disulfides can be driven solely by the thioredoxin pathway. Our results highlight the similarity between the pathways for reduction of disulfides in the bacterial periplasm and the mammalian ER.

## RESULTS

### The reduction of ER-localised disulfides requires an ER membrane component

**Fig. 1.**
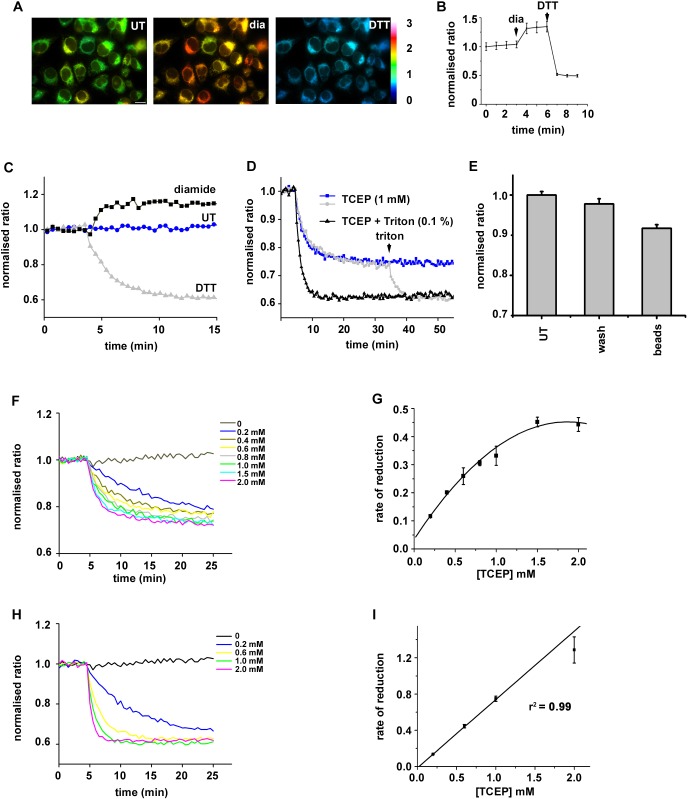
**Reduction of ER-localised disulfides by TCEP requires a membrane component.** A stable cell-line expressing ER-SFGFP-iE was evaluated by live-cell imaging and was responsive to gross changes in redox conditions. (A) Fluorescent images of untreated, diamide- (1 mM) or DTT-treated (10 mM) cells. Fluorescence ratio changes are visualised using false colours, with a more reducing ratio in blue and a more oxidising in yellow. (B) Normalised fluorescent ratios of individual cells (*n*=37) are averaged (mean±s.d.) and changes observed over time by the sequential addition of diamide (1 mM) and DTT (10 mM). (C) Normalised fluorescence ratio changes in microsomal membranes isolated from the cell line expressing ER-SFGFP-iE, followed using a plate reader. An increase in ratio is seen following diamide (1 mM) addition whereas a decrease is seen following DTT (1 mM) addition. The ER-SFGFP-iE in untreated cells is, therefore, neither fully oxidised nor reduced. The experiment was repeated four times (*n*=5). (D) Changes in normalised fluorescence ratio following the addition of impermeable TCEP in the absence (blue) or presence (black) of Triton X-100. After 35 min, Triton X-100 (0.1% v/v) was added to solubilise the intact microsomes. Note the slower rate of reduction and the lack of full reduction with TCEP unless the membranes are solubilised. The experiment was repeated twice (*n*=3). (E) ER-SFGFP-iE is reduced by TCEP immobilised on agarose beads, further confirming that the reduction is not direct. The experiment was carried out in triplicate with average (mean±s.d.) indicated for untreated (UT), buffer preincubated with TCEP beads (wash) and TCEP beads (beads). (F-I) The change in normalised fluorescent ratio was followed at various TCEP concentrations as indicated either without (F) or with (H) detergent solubilisation of microsomes. The rate of reduction at each TECP concentration was calculated in triplicate with the average (mean±s.d.) plotted versus the TCEP concentration (G,I).

To follow the reduction of disulfide bonds within the ER lumen, we created a HT1080 stable cell line expressing a version of roGFP that can act as a reporter of disulfide formation within the ER of mammalian cells. To improve the folding and stability of roGFP, we included the superfolder mutations as described previously (Hoseki et al., 2016), but using an ER targeted roGFP1-iE rather than roGFP1-iL. The resulting cell line demonstrated bright ER-localised fluorescence that was responsive to changes in both oxidation and reduction, making it an ideal reporter for changes in ER redox state (Fig. 1A,B). In addition, there was an absence of light-induced fluorescence changes, an effect that compromised the use of the roGFP1-iL variant (van Lith et al., 2011). The variant of roGFP was designated ER-SFGFP-iE. We isolated microsomal vesicles from the ER-SFGFP1-iE cell line and were able to follow changes to ER redox status over time using a plate reader (Fig. 1C). We established that the microsomes were sensitive to both reduction with dithiothreitol (DTT) and oxidation with diamide, indicating that the reporter was neither fully oxidised nor reduced following isolation. Reduction with membrane-permeable DTT was rapid, reaching completion within 10 min. When the membrane-impermeable reducing agent tris-(2-carboxyethyl)phosphine (TCEP) was added to microsomes containing ER-SFGFP-iE, reduction still occurred but much slower than if the microsomes were solubilised prior to TCEP addition (Fig. 1D, blue versus black). Reduction in intact microsomes did not reach completion unless detergent was added to solubilise the ER membrane (see from 35 min in Fig. 1D). To ensure the TCEP was not directly reducing ER-SFGFP-iE, we carried out a similar experiment but used TCEP immobilised on agarose beads. ER-SFGFP-iE was reduced significantly (*P*=0.00001) by immobilised TCEP agarose beads but not by buffer pre-incubated with TCEP beads (*P*=0.03) (Fig. 1E). These results strongly suggest the indirect reduction of ER-SFGFP-iE by TCEP when the microsomal membrane is intact.

The lack of full reduction of ER-SFGFP-iE by TCEP suggests that the reaction had reached saturation. To evaluate this possibility further we determined the rate of reduction with a range of TCEP concentrations (Fig. 1F,G). The results show that the rate of reduction does indeed reach saturation at concentrations of TCEP over 1 mM, suggesting a facilitated process. To show that TCEP can reduce ER-SFGFP-iE directly and fully we determined the rate of reduction with a range of TCEP concentrations after solubilising the ER membrane (Fig. 1H,I). Under these conditions the rate did not reach saturation, suggesting a diffusion-limited process. These results with the membrane-impermeable reducing agent suggest the indirect reduction of an ER-localised disulfide mediated via an ER membrane component.

### The thioredoxin reduction pathway is sufficient to reduce ER-localised proteins via a membrane protein

**Fig. 2.**
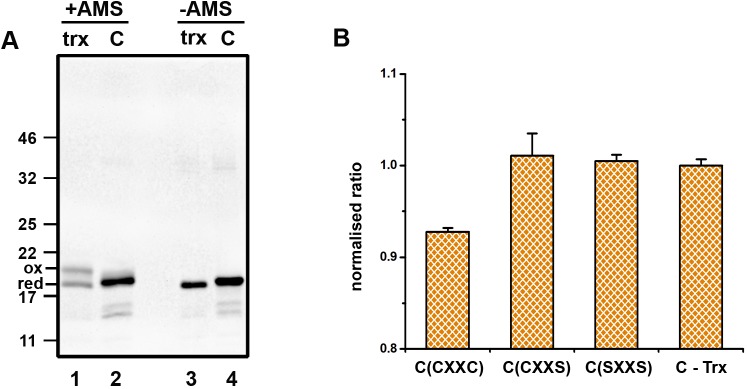
**Thioredoxin-dependent reduction of ER-localised disulfides.** (A) The redox status of Trx1 (trx) was determined by AMS differential alkylation either before (lane 1) or after (lane 2) incubation with TrxR1 and a NADPH recycling system (C). Samples not treated with AMS are shown in lanes 3 and 4. The experiment was carried out once. (B) The normalised fluorescence ratio of ER-SFGFP-iE was measured following incubation with the reduction system without Trx1 (C-Trx) or with the TrxR1 system containing wild-type Trx1 (CXXC) or active site mutants CXXS or SXXS. The experiment was repeated twice (*n*=3) with the average (mean±s.d.) presented.

To evaluate the cytosolic components required to ensure reduction of ER-localised disulfides, we attempted to reconstitute the pathway with purified components. We reasoned that a source of NADPH would be required as well as TrxR1 and Trx1. To ensure efficient recycling of NADPH, we included glucose 6 phosphate (G6P) as well as G6P dehydrogenase. To demonstrate that the system could maintain Trx1 in its reduced state, we determined the redox status of purified Trx1 before and after incubation with the recycling system. Redox status was evaluated following modification with 4-acetamido-4′-malemidylstilbene-2,2′-disulfonic acid (AMS), which increases the protein mass and thus slows its electrophoretic mobility. Prior to incubation, Trx1 was present as a mixture of both reduced and oxidised protein (Fig. 2A, lane 1). After incubation with TrxR1 and the NADPH recycling system, Trx1 was fully reduced (Fig. 2A, lane 2). When our microsomes containing ER-SFGFP-iE were incubated with the TrxR1 reduction system, roGFP became more reduced, an effect that was dependent on the inclusion of Trx1 as no reduction occurred in the presence of TrxR1 and the NADPH recycling system alone (Fig. 2B, C-Trx). No reduction of roGFP was observed when active site mutants of Trx1 (CXXS or SXXS) were used in place of wild-type protein (CXXC). These results demonstrate that the minimal requirement for reduction of ER-localised roGFP is the thioredoxin reduction system and that disulfide exchange is mediated through the Trx1 CXXC active site motif. As the reduction was Trx1 dependent and Trx1 is membrane impermeable, it is highly likely that an ER membrane component is required to transfer the reducing equivalents from Trx1 to roGFP.

The experiments with roGFP provide us with a useful way to follow reduction of a specific disulfide within the ER lumen by components added outside the vesicles; however, this protein is not normally present in the ER. Hence, we evaluated the redox status of ERp57, a member of the PDI family, to determine whether the thioredoxin reduction system could also reduce an endogenous ER protein that has previously been shown to be required for the correct folding of glycoproteins (Jessop et al., 2007).

**Fig. 3.**
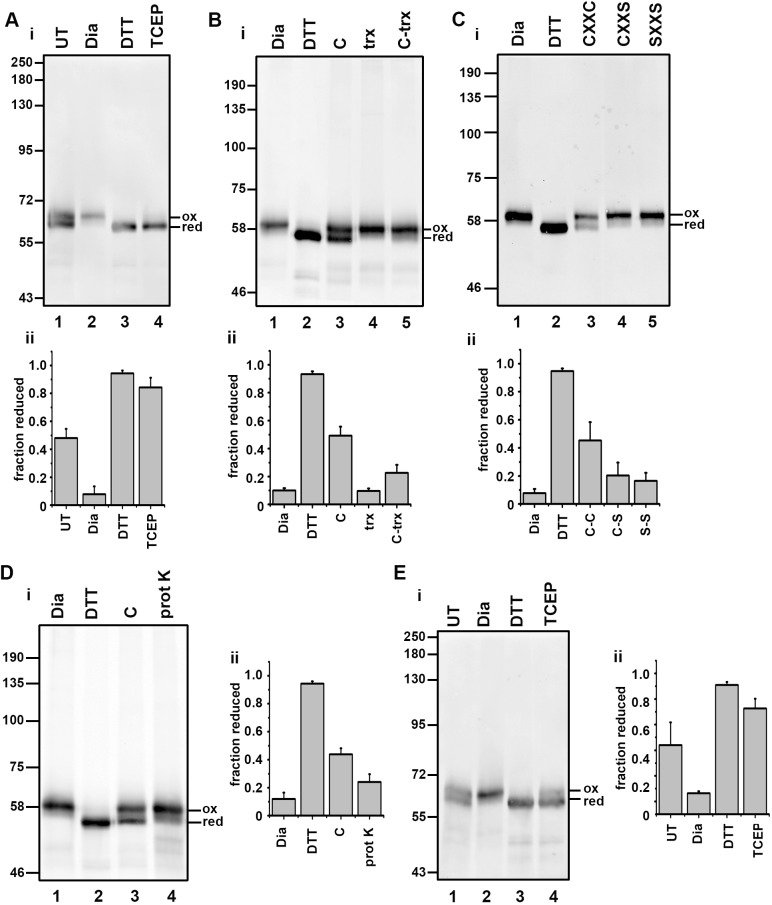
**Thioredoxin-dependent reduction of ERp57 requires a membrane protein.** (A) The redox status of ER-localised ERp57 was determined following differential alkylation with AMS. (Ai) In untreated SP cells, ERp57 exists as a mixture of reduced and oxidised forms (lane 1) and becomes fully oxidised (lane 2) or fully reduced (lane 3) following the addition of diamide (Dia) or DTT, respectively. Partial reduction of ERp57 also occurs following incubation of fully oxidised ERp57 with the membrane-impermeable reductant TCEP (lane 4). (Aii) Quantification of the fraction reduced in untreated SP cells (UT), SP cells treated with diamide, DTT or TCEP. The experiment was repeated twice (*n*=3). (B) Thioredoxin-dependent reduction of ER-localised ERp57. (Bi) SP cells were treated with diamide before incubation with either the complete TrxR1 reduction system (C; lane 3) or without (C–trx; lane 5) or with Trx1 alone (trx; lane 4). ERp57 was partially reduced only by the complete system. (Bii) Quantification of the fraction reduced in SP cells treated as for Bi. The experiment was repeated twice (*n*=3). (C) The reduction of ERp57 by the TrxR1 system needs Trx1 with an intact active site. (Ci) Neither of the active site mutants CXXS (lane 4) or SXXS (lane 5) can substitute for wild-type (CXXC) Trx1. (Cii) Quantification of the fraction reduced in SP cells treated as for Ci. The experiment was repeated twice (*n*=3). (Di) The reduction of ER-localised ERp57 by the TrxR1 system is sensitive to prior treatment of the SP cells with proteinase K (lane 4). (Dii) Quantification of the fraction reduced in SP cells treated with diamide, DTT or a TrxR1 reduction system either before (C) or after (protK) treatment of the SP cells with proteinase K. The experiment was repeated twice (*n*=3). (Ei) SP cells were treated with proteinase K in the absence of further treatment (UT) or after treatment with diamide, DTT or TCEP. The reduction of ER-localised ERp57 by TCEP is not prevented by prior treatment of the SP cells with proteinase K (lane 4). (Eii) Quantification of the fraction reduced in SP cells treated as for Ei. The experiment was repeated twice (*n*=3). All graphs show mean values; error bars indicate s.d.

We used the approach described for AMS (Jessop and Bulleid, 2004) to probe the redox status of ERp57 in SP cells isolated from a cell line stably transfected with V5-tagged ERp57 (Jessop et al., 2007). Samples were either untreated or treated with the oxidising agent diamide, with the reducing agent DTT or with the membrane-impermeable TCEP (Fig. 3Ai,ii). Treatment with *N*-ethylmaleimide (NEM) blocks any free thiols and subsequent reduction, and alkylation with AMS reduces the mobility of the protein. Proteins that contain disulfides in the original SP cells, therefore, show reduced mobility on SDS-PAGE. Following this procedure, ERp57 in untreated SP cells migrated as two species representing the oxidised and reduced forms, by comparison with the diamide- or DTT-treated samples (Fig. 3Ai, lanes 1-3). After incubation with TCEP most of the ER-localised ERp57 was reduced, although not completely (Fig. 3Ai, lane 4), a result that mirrors the incomplete reduction of roGFP and demonstrates that TCEP can substitute for a cytosolic reduction system.

To evaluate the ability of the thioredoxin pathway to reduce ER-localised ERp57, we first fully oxidised SP cells with diamide, removed diamide by re-isolating the SP cells and incubated them with the complete TrxR1 reduction system, just recombinant Trx1 or the reduction system without Trx1 (Fig. 3Bi,ii). Partial reduction of fully oxidised ERp57 occurred in the presence of the complete system (Fig. 3Bi, lane 3) but did not occur with just Trx1 alone (Fig. 3Bi, lane 4) indicating a requirement for the recycling system. The reduction of ERp57 was dependent on Trx1 (Fig. 3Bi, lane 5) as was the case with roGFP. In addition, the active site mutants of Trx1 lacking one or both cysteines within the CXXC motif were unable to reduce ERp57 (Fig. 3Ci,ii; lanes 4 and 5). These results demonstrate that ER-localised ERp57 can be reduced following oxidation by the cytosolic reduction pathway and that this reduction is dependent on thioredoxin.

To further characterise the membrane component required for reduction, we carried out a proteinase K digestion of our SP cells prior to assay for ERp57 reduction (Fig. 3Di,ii). We surmised that if a membrane protein was involved then the transfer of reducing equivalents across the membrane might be sensitive to proteolysis. Proteinase K digestion prevented the reduction of oxidised ERp57 by the reconstituted reduction system (Fig. 3Di, lane 4), suggesting the requirement for a membrane protein whose cytosolic domain is sensitive to digestion. We also determined whether the reduction of ERp57 by TCEP was sensitive to proteinase K treatment of the SP cells (Fig. 3Ei,ii). TCEP was still able to reduce oxidised ERp57 (Fig. 3Ei, lane 4), suggesting that either the membrane protein involved can be reduced efficiently by TCEP without its protease-sensitive cytosolic domain or that TCEP acts via a component separate from the thioredoxin-dependent pathway.

### The thioredoxin reduction pathway is sufficient to reduce non-native disulfides in nascent chains exposed to the ER lumen

**Fig. 4.**
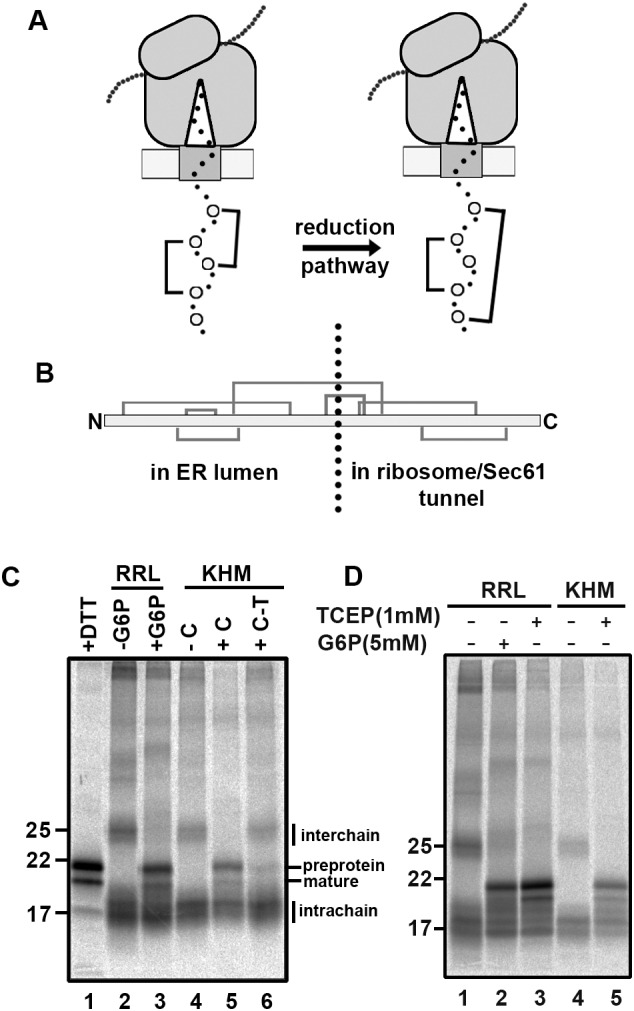
**Thioredoxin-dependent post-translational rearrangement of ER-localised nascent chain disulfides.** (A) Cartoon depicting the rearrangement of nascent chain disulfides in a stalled translocation intermediate following the addition of a cytosol-localised reduction system. (B) Schematic of the disulfide pattern within the disintegrin domain of ADAM10. The stalled intermediate used in subsequent experiments has the indicated disulfide-forming cysteines in the ER lumen. (C) Translations carried out in the presence of DTT (5 mM) gave rise to distinct translation products that migrate with the sizes of the pre-protein and mature protein as indicated (lane 1). Translations were carried out in the absence of added G6P and SP cells subsequently incubated post-translationally in a reticulocyte lysate (RRL) in the absence (lane 2) or presence (lane 3) of G6P. SP cells were isolated from the translations and resuspended in KHM buffer alone (−C) (lane 4). Note the rearrangement of nascent chain disulfides when the TrxR1 system is included in the incubation (lane 5) (+C) and the dependence of this rearrangement on the presence of Trx1 (lane 6) (C-T). The experiment was repeated once (*n*=2). (D) Similarly to the TrxR1 system, TCEP brings about the rearrangement of nascent chain disulfides when added to SP cells post-translationally either in the presence of RRL (lane 3) or in KHM buffer alone (lane 5). The experiment was repeated once (*n*=2).

We previously demonstrated that non-native disulfides formed during the synthesis of β1 integrin require a cytosolic reductive pathway to ensure their isomerisation to the correct disulfides (Poet et al., 2017). To evaluate the ability of our reconstituted pathway to isomerise non-native disulfides, we generated stalled translocation intermediates of a protein that forms non-native disulfides following *in vitro* translation in the presence of SP cells (Robinson et al., 2020). By isolating SP cells containing these intermediates, we were then able to follow the rearrangement of disulfides post-translationally in the presence or absence of a TrxR1 reduction system (Fig. 4A). We generated a stalled translocation intermediate of the disintegrin domain of ADAM10 by translating an RNA transcript lacking a stop codon. The length of the synthesised chain is sufficient to allow exposure of several cysteine residues in the ER lumen (Fig. 4B) that have the potential to form disulfides. Translations were performed using a rabbit reticulocyte lysate supplemented with SP cells to study folding in the ER (Wilson et al., 1995). All samples were treated with NEM on completion to irreversibly modify thiols and freeze the disulfide status of the samples for downstream processing. When translations were carried out in the presence of a reducing agent (DTT) and the samples separated under non-reducing conditions, two products of approximately 18 and 20 kDa were synthesised (Fig. 4C, lane 1). These products correspond to the translocated, signal sequence cleaved chain and untranslocated preprotein (Robinson et al., 2020). When the translations were carried out in the absence of added G6P and a post-translational incubation carried out in the absence of G6P, a diffuse pattern emerged with the translation products forming both intra- and interchain disulfides (Fig. 4C, lane 2). When G6P was added post-translationally and samples incubated for 60 min, more distinct products were formed indicative of discrete disulfide-bonded species (Fig. 4C, lane 3). When SP cells were isolated from translations carried out in the absence of G6P and incubated in buffer alone, no change to the banding pattern was observed (Fig. 4C, lane 4). However, when the TrxR1 reduction system was added to the isolated SP cells, rearrangement of disulfides was observed and resulted in more distinct disulfide-bonded species (Fig. 4C, lane 5). This change to the banding pattern to more discrete disulfide-bonded species did not occur when the isolated SP cells were incubated in the TrxR1 reductive pathway lacking Trx1 (Fig. 4C, lane 6). We also demonstrated that TCEP could substitute for G6P when added to translations or to isolated SP cells post-translationally, giving rise to discrete disulfide-bonded species (Fig. 4D, lanes 3 and 5). We have previously shown that the disulfide-bonded products formed from this ADAM10 construct are present in nascent chains translocated into the ER lumen (Robinson et al., 2020). Taken together, these results show that a source of non-membrane-permeable reducing agent can resolve the non-native disulfides formed in nascent chains within the ER lumen.

### The thioredoxin reduction pathway reduces disulfides in several secretory and ER resident proteins

**Fig. 5.**
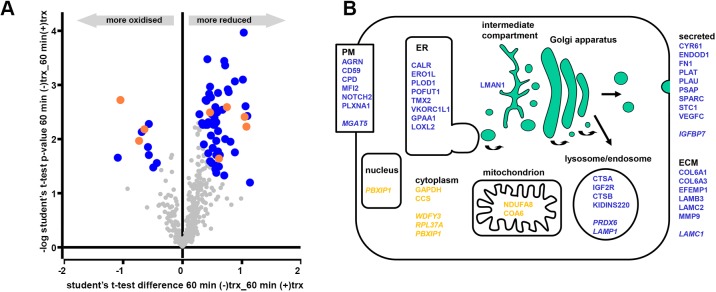
**Evaluation of the changes in the redox status of microsomal proteins following incubation with the TrxR1 system.** (A) Volcano plot depicting proteins with significant changes to their redox status where the modified cysteine is localised to the ER (blue) or the cytosol (orange). Proteins identified by mass spectrometry whose redox status does not change significantly are also indicated (grey). (B) Cartoon illustrating the final cellular location of proteins whose redox status changes significantly upon incubation of microsomes with the TrxR1 system. Proteins that enter the secretory pathway are in blue whereas those that do not enter the ER are in yellow. Proteins that become more reduced are in normal text and those that become more oxidised are in italics. Note that PBXIP1 associates with the cytoskeleton and is mainly in the cytosol but it does shuttle to the nucleus to act in addition to its role as a soluble transcription factor (Abramovich et al., 2002).

The experiments with roGFP and ERp57 are dependent on the reduction of a reversible disulfide that is solvent accessible in the final protein structure. To determine whether our reconstituted reductive system could reduce other disulfides within secretory or membrane proteins, we carried out redox proteomics. Essentially, microsomes were incubated with the TrxR1 system in either the presence or absence of Trx1 for 60 min and the reversibly oxidised thiol groups labelled with heavy iodoacetamide. The samples were prepared for liquid chromatography with tandem mass spectrometry (LC/MS/MS) analysis by trypsin digestion followed by tandem mass tagging to enable quantification of the changes in the cysteine oxidation levels between experimental conditions. The resulting data were analysed by Perseus software and are presented as a volcano plot indicating the statistically significant changes to the reversibly oxidised cysteine residues upon incubation with Trx1 (Fig. 5A). The redox status of several peptides changed during the incubation; specifically, there was a subset of cysteines whose oxidised status decreased (became more reduced) in the reactions containing Trx1, with the majority of the corresponding proteins being located in the ER lumen (Fig. 5A, blue circles). Notably, the regulatory cysteine (Cys131) in Ero1α was reduced (Appenzeller-Herzog et al., 2008; Baker et al., 2008) as was the cysteine involved in recycling VKORC1L1 (Cys50) (Tie et al., 2014). Changes to the redox status of a few cytosolic and mitochondrial proteins also occurred (Fig. 5A, orange circles), which probably co-purified with our microsomal vesicles. The identity of all the proteins identified whose redox status changed along with their ultimate subcellular location is as illustrated (Fig. 5B) or detailed separately (Table 1). The results demonstrate that several thiols within endogenous proteins that are synthesised at the ER, including thiols that form structural and regulatory disulfides, can be reduced by the thioredoxin reduction system. The target proteins were localised to the ER lumen and the TrxR1 reduction system was membrane impermeable, illustrating the requirement to transfer reducing equivalents across the membrane.

**Table 1.**
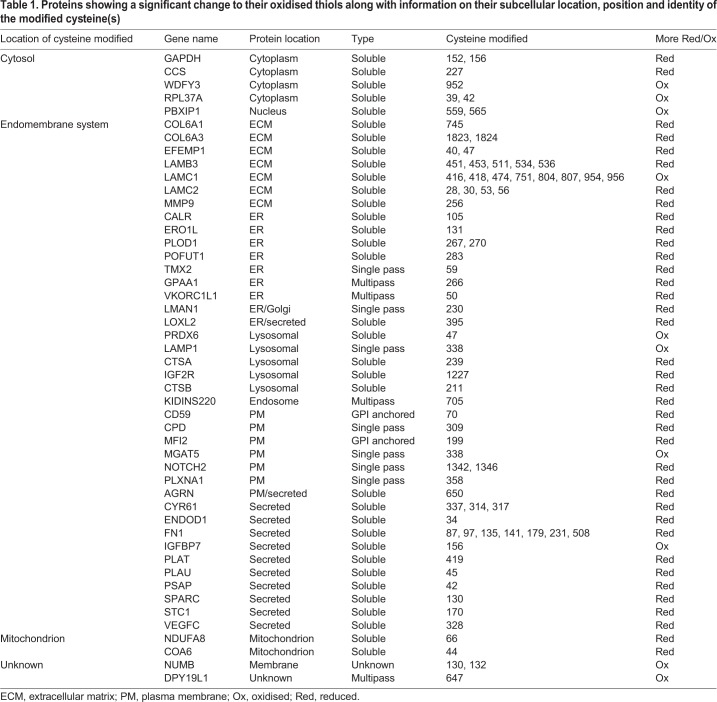
**Proteins showing a significant change to their oxidised thiols along with information on their subcellular location, position and identity of the modified cysteine(s)**

## DISCUSSION

Our previous work provided the first indication that a cytosolic reductive pathway was required to ensure correct disulfide formation within the ER (Poet et al., 2017). Here, we demonstrate that the thioredoxin pathway is sufficient to provide the reducing equivalents to ensure the reduction of regulatory and non-native disulfides within ER proteins. We have successfully reconstituted the reduction of ER disulfides using a microsomal system in the presence of purified components to demonstrate that the thioredoxin pathway can drive the reduction of disulfides in several ER-localised soluble and membrane proteins. In addition, we show that the pathway is sufficient to facilitate the reduction of disulfides formed in nascent chains entering the ER lumen but does not prevent disulfide re-formation, a requirement for correct isomerisation of non-native to native disulfides. Importantly, we show that a membrane protein is required to facilitate the transfer of reductant across the ER membrane, presumably via a disulfide exchange mechanism as demonstrated for the transfer of disulfides across the bacterial plasma membrane by DsbD (Cho et al., 2007).

The absolute requirement for the presence of thioredoxin in our reconstituted system rules out a role for an NADPH-dependent ER-localised reductase. Previously, it has been postulated that an ER glutathione or thioredoxin reductase could utilise the ER pool of NADPH to drive the reduction of disulfides (Bulleid and Ellgaard, 2011). As there is an ER-localised hexose 6-phosphate dehydrogenase (Lavery et al., 2006), G6P might enter the ER via the G6P-transporter, be metabolised to phosphogluconolactone and in the process regenerate the ER pool of NADPH. Hence, the G6P effect we previously observed during our *in vitro* translations might be the result of a process independent of the thioredoxin pathway. This is clearly not the case for the reduction of roGFP, ERp57, disulfides in nascent chains and the range of proteins identified from the redox proteomics whose redox state change depends on the presence of thioredoxin.

The reductive pathway driven by cytosolic thioredoxin is versatile in terms of its target disulfides in the ER lumen. These disulfides included those present within folded proteins such as roGFP, in proteins undergoing folding during translocation and in proteins that require reduction to maintain their activity. Such versatility suggests a broad substrate specificity of the terminal enzyme involved in catalysing reduction. Changes to the redox status of ERp57 would suggest that this member of the PDI family could act as a reductase, as suggested previously for newly synthesised glycoproteins (Jessop et al., 2007) to which it is targeted via its interaction with calnexin or calreticulin (Oliver et al., 1997). It is also likely that ERdj5 is reduced by the cytosolic reductive pathway and targeted to substrate proteins through its interaction with BiP (Cunnea et al., 2003). Our identification of one of the regulatory disulfides within Ero1α (Baker et al., 2008) as a target for the cytosolic reductive pathway suggests that this disulfide can be reduced by one of the PDI family members, as demonstrated previously using purified proteins (Shepherd et al., 2014). Whether the ER membrane protein responsible for transfer of reducing equivalents directly reduces the PDI reductase remains to be established.

The role of thioredoxin as reductant of the ER membrane protein can be replaced with the membrane-impermeable reducing agent TCEP. However, in contrast to thioredoxin-mediated process, reduction of ER proteins via TCEP was shown to be insensitive to protease digestion, suggesting that this reagent can reduce the ER membrane protein in the absence of a protease-sensitive cytosolic domain. This result probably reflects the different requirements for reduction of the membrane protein disulfide by thioredoxin and TCEP. The mechanism underlying recognition and reduction of substrates by thioredoxin is not completely understood given its wide range of substrate specificity. However, it has been demonstrated that noncovalent interactions prior to disulfide reduction are important, with recognition being due to the conformational restriction afforded by the oxidised substrate (Palde and Carroll, 2015). Hence, the proteolytic digestion of a protein domain or loop might prevent thioredoxin interacting with the membrane protein, thereby inhibiting activity. A chemical reductant such as TCEP has no requirement for such noncovalent interactions and only requires the target disulfide to be solvent accessible.

The identity of the membrane protein involved in connecting the cytosolic thioredoxin couple with the ER remains unknown. One resident ER membrane protein whose depletion leads to a more oxidising ER and a specific defect in correct disulfide formation in lipoprotein lipase is lipase maturation factor 1 (LMF1) (Roberts et al., 2018). This protein contains several thiols in its cytosolic loops, transmembrane regions and lumenal domain that are required for activity and could be involved in disulfide exchange. Unfortunately, we did not identify LMF1 in our proteomics data, so we could not identify any changes to its redox status. Hence, LMF1 remains a potential candidate for shuttling disulfides across the ER membrane. Of the 41 microsomal proteins whose redox status changed during incubation with the TrxR1 system, four are multipass membrane proteins with VKORC1L1 and GPAA1 being known resident ER proteins. GPAA1 is a subunit of the GPI-anchor transamidase (Ohishi et al., 2000) whereas VKORC1L1 is a paralogue of vitamin K epoxide hydrolase with a potential function in vitamin K-dependent carboxylation (Tie et al., 2013). Whether these proteins are directly involved in disulfide exchange across the ER membrane is a focus of future studies.

## MATERIALS AND METHODS

### Generation of stable cell line expressing ER-SFGFP-iE

HT1080 cells were transfected with ER-SFGFP-iE using MegaTran 1.0 (OriGene, Cambridge, UK) following the manufacturer's protocol. Various dilutions (1:10 to 1:10,000) of transfected HT1080 cells were plated on 15 cm^2^ dishes. Cells were grown in selective medium containing G418 (1 mg/ml) until colonies appeared. Single colonies were picked and transferred to 12-well plates (one colony per well) using trypsin-soaked Scienceware cloning discs (Sigma, Dorset, UK). Cells were grown until confluent and then transferred to 25 cm^2^ flasks. The presence of ER-SFGFP-iE was confirmed by western blot using rabbit anti-GFP antibody (Santa Cruz, California, USA; cat# sc-8334, 1:200 dilution). All cell lines were regularly tested for mycoplasma infection.

### Live cell microscopy and image analysis

HT1080 cells stably expressing ER-SFGFP-iE were seeded on coverslips. The cells were washed with buffer [HEPES (20 mM) pH 7.4, NaCl (130 mM), KCl (5 mM), CaCl_2_ (1 mM), MgCl_2_ (1 mM), D-glucose (10 mM)] before imaging on a Zeiss Axio Observer A1 inverted microscope equipped with a 40× FLUAR lens (Zeiss, Oberkochen, Germany). The cells were sequentially excited by 385 and 470 nm LEDs with fluorescence detection through a T495 lpxr beamsplitter (Chroma, Vermont). Images were captured at 30 s intervals and analysed with AxioVision 4.8 software. The 385/470 nm ratio images of untreated, diamide-treated (1 mM) and DTT-treated (10 mM) cells were false-coloured using a rainbow look-up table. The 385/470 nm ratio changes over time were quantified by defining regions of interest for individual cells and plotted as the average ratio with standard deviation.

### Preparation of microsomal membranes

Cells were cultured using cell culture roller bottles (1700 cm^2^) (Greiner Bio-One). Confluent cells were detached from the roller bottle by incubation with 100 ml PBS containing EDTA (1 mM) for 15 min. Cells were pelleted, washed three times in HEPES–KOH (35 mM) buffer at pH 7.5 containing NaCl (140 mM) and glucose (11 mM) followed by washing once in extraction [HEPES–KOH (20 mM) pH 7.5, KOAc (135 mM), KCl (30 mM), MgOAc (1.65 mM)]. An equal volume of extraction buffer to the cell volume was used for resuspension. The cells were disrupted in a Mini-Bomb Cell disruption chamber (Kontes) using a nitrogen pressure of 1.0 MPa for 30 min. Cell homogenates were centrifuged at 6000×***g*** for 5 min at 4°C and the post-nuclear supernatant retained. The supernatant was centrifuged at 50,000×***g*** (Optima Max-XP Ultracentrifuge, Beckman Coulter) for 15 min at 4°C to pellet the membrane fraction. The membrane pellet was resuspended in buffer A [Tris-HCl (50 mM) pH 7.4, sucrose (0.25 M), KCl (50 mM), MgOAc (6 mM), EDTA (1 mM)] to give an *A*_280_ value of 50 units/µl (the absorbance was determined in water in the presence of 1% SDS). Aliquots of the microsomal membranes were frozen in liquid nitrogen and stored at −80°C.

### Measurement of changes to the redox state of microsomal ER-SFGFP-iE

Microsomes were pipetted into Dulbecco's phosphate-buffered saline (PBS) (1.5 μl microsomes in 48.5 µl PBS) in the absence or presence of 0.1% Triton X-100 (Thermo Fisher). The solution was added into a CELLSTA 96-Well Polystyrene Flat Bottom Cell Culture Microplate (Greiner Bio-One) (final volume 50 μl per well; three wells per condition). Fluorescence intensities at 520 nm were measured following excitation at 390 nm and 460 nm excitations wavelengths using PHERAstar FS microplate reader (BMG Labtech). After 10 min of measurement to obtain a baseline, DTT, diamide or different concentrations of tris-(2-carboxyethyl)phosphine (TCEP) (Thermo) were injected into the wells. Fluorescence intensities were recorded for up to 50 min. In some experiments, the same amount of microsomes were untreated or treated either with immobilized TCEP Disulfide Reducing Gel beads (Thermo) that had been washed twice with PBS to remove soluble TCEP or with PBS that had been preincubated with TCEP beads for 60 min. The samples were incubated for 60 min and then transferred into microplate wells to measure fluorescence intensity at 520 nm following excitation at 390 and 460 nm.

To calculate the rate of change of the redox status of ER-SFGFP-iE at different TCEP concentrations, microsomes were treated with various concentrations of TCEP in the absence or presence of Triton X-100 (0.1% v/v). The fluorescence ratio (390/460 nm) change was followed over time after addition of TCEP and the resulting time course fitted to an exponential decay function [*y*=-*A*(e^-k(t-t0)^-1)+*c*] to calculate the rate (*k*), where *t*_0_ is time of addition of TCEP, *c* the initial fluorescence ratio and *A* the difference in fluorescence ratio between *t*_0_ and *t*. The rate was then plotted against TCEP concentration. Measurement of the rate of reduction of ER-SFGFP-iE at different concentrations of TCEP was performed in triplicate.

### Recombinant protein purification

Recombinant human thioredoxins (Trx1), wild type and mutants, were expressed and purified from *Escherichia coli* BL21-DE3 cells as described previously (Schwertassek et al., 2007). Purification was by successive HisTrap affinity and size exclusion (Superdex 200 10/300) chromatography (GE Healthcare). The protein concentration was determined following OD_280nm_ measurement and calculated using an extinction coefficient of 12.49 mM^−1^ cm^−1^. The purified protein aliquots were flash frozen in liquid nitrogen and stored −80°C.

### Determining the redox state of Trx1

Purified thioredoxin (25 µM) was incubated in KHM buffer [HEPES (20 mM) pH 7.2 containing KOAc (110 mM) and MgOAc (2 mM)] at 30°C for 60 min in the absence or presence of NADPH (1 mM), human TrxR1 (16.25 nM; IMCO), G6P (1.25 mM) and G6PDH (10 U/ml). The reaction was stopped by the addition of NEM (20 mM). Samples were incubated with streptavidin agarose resin (Thermo) in isolation buffer (IB) [Tris-HCl (50 mM) pH 7.5, Triton (1% v/v), NaCl (150 mM), EDTA (2 mM), PMSF (0.5 mM), sodium azide (0.02% w/v)] at 4°C for 60 min. The streptavidin beads were pelleted by centrifugation and washed three times in isolation buffer to remove unbounded proteins. Trx1 was eluted from the streptavidin beads by incubating at room temperature (RT) for 15 min with PBS containing SDS (2% w/v) and biotin (3 mM). The samples were boiled at 105°C for 15 min and incubated with TCEP (10 mM) at RT for 10 min to break existing disulfides, followed by a 1.5 h incubation with AMS (20 mM; Invitrogen) in the dark to alkylate free thiols. The samples were separated by SDS-PAGE and a western blot performed using Streptavidin Protein DyLight 800 (ThermoFisher, cat#21851; 1:5000 dilution).

### Preparation of SP cells

SP cells were prepared as described previously (Wilson et al., 1995). Following digitonin treatment, cells were resuspended in KHM buffer containing CaCl_2_ (1 mM) and treated with *Staphylocossus aureus* nuclease (150 U/ml; Calbiochem) at RT for 12 min to remove endogenous mRNA. The reaction was stopped with EGTA (4.5 mM; Sigma). SP cells were pelleted by centrifugation and resuspended in 100 µl KHM buffer to be used in translation reactions. For ERp57 redox state determination experiments, SP cells were prepared from cells expressing V5-tagged ERp57 (Jessop et al., 2007); nuclease treatment was not included during the preparation.

### Determining the redox state of ER-localised ERp57

The redox state of ERp57 in SP cells was determined as described previously (Jessop and Bulleid, 2004). SP cells were either untreated or treated with diamide (10 mM) at RT for 10 min. Samples were washed twice with 500 µl KHM buffer to remove diamide. Oxidised SP cells were incubated at 30°C for 60 min in KHM buffer with or without DTT or with the purified reductive pathway components NADPH (1 mM), human Trx1 (25 µM), human TrxR1 (16.25 nM), G6P (1.25 mM) and G6PDH (10 U/ml). The SP cells were pelleted by centrifugation at 14,800×***g*** for 1 min and then incubated with 500 µl PBS containing NEM (25 mM) to block free thiols. The samples were washed twice with 1 ml PBS to remove excess NEM. The cells were lysed on ice with lysis buffer [Tris-HCl (50 mM), NaCl (150 mM), EDTA (2 mM), PMSF (0.5 mM), Triton X-100 (1% v/v)] for 10 min. The lysate was centrifuged at 14,800×***g*** at 4°C for 10 min. The supernatant was removed and boiled with SDS (2% w/v; VWR chemicals) to denature the protein. Denatured samples were incubated at RT with TCEP (10 mM) to break existing disulfide bonds, followed by a 1.5 h incubation with AMS (20 mM; Invitrogen) in the dark. The samples were separated by SDS-PAGE and a western blot performed with antibody to V5-tag to detect different redox states of ERp57.

### Western blotting

Following SDS-PAGE, proteins were transferred to nitrocellulose blotting membrane (GE Healthcare). The membrane was blocked with 3% w/v dried skimmed milk (Marvel) in TBST buffer [Tris-HCl pH 8.0 (150 mM), NaCl (150 mM), Tween-20 (0.5% v/v)] for 1 h. The membrane was incubated in TBST including V5-antibody (Invitrogen, cat#R960-25; 1:10,000 dilution) for 1 h. After primary antibody incubation, the membrane was incubated in 5 ml TBST with secondary antibody (Fisher Scientific, cat #10751195; 1:10,000 dilution) in the dark for 45-60 min. The membrane was scanned using a Li-Cor Odyssey 9260 Imager. Quantification of gel intensity was by ImageJ (National Institutes of Health, Bethesda, MD); experiments were carried out three times. Values quoted are averages, with error bars depicting standard deviation.

### Proteinase K treatment

SP cells were incubated on ice with proteinase K (20 µg/ml; Roche) for 25 min in the presence of CaCl_2_ (10 mM). Proteinase K was inactivated by incubation with PMSF (0.5 mM). Proteinase K and PMSF were removed by washing twice with KHM buffer.

### Preparation of ADAM10 domain construct and *in vitro* translation

The human ADAM10 construct codes for residues 456-550 and contains the human-β2 M signal sequence residues (1-20). The generation of this construct and its transcription and translation were described previously (Robinson et al., 2020). Essentially, plasmid DNA was used as a template for the polymerase chain reaction (PCR) using an appropriate forward primer that adds a T7 promoter and a reverse primer that lacks a stop codon. The PCR product was transcribed into an RNA template using T7 RNA polymerase. Translations were performed using the Flexi Rabbit Reticulocyte Lysate System (Promega) supplemented with DTT (10 mM) or purified water but in the absence of added G6P. SP cells were added to a concentration of ∼10^5^ cells per 25 μl translation reaction. Following assembly of components, translation reactions were incubated at 30°C for 15 min. For post-translational assays, G6P (5 mM) was added following translation or the SP cells were isolated by centrifugation and resuspended in KHM buffer in the presence or absence of a TrxR1 reduction system [NADPH (1 mM), human Trx1 (25 µM), human TrxR1 (16.25 nM), G6P (1.25 mM) and G6PDH (1 U) or TCEP (1 mM)]. An additional incubation was carried out in the presence of the TrxR1 reduction system without Trx1. The reactions were stopped after a 60 min incubation by the addition of NEM (final concentration 25 mM) prior to immunoisolation, SDS-PAGE and phosphorimage analysis.

### Immunoisolation

SP cells from the translation reactions were resuspended in 1 ml IB containing 50 µl 10% Protein A Sepharose FF Resin (Generon). After 30 min incubation, the beads were isolated by centrifugation at 16,000 ***g*** for 30 s. The supernatant was transferred into a new tube containing 10% Protein A Sepharose (50 µl). Anti-V5 antibody (Invitrogen, cat #10751195; 1 µl) was added and the suspension incubated at 4°C overnight. The beads were pelleted by centrifugation and washed three times with 1 ml IB buffer. The beads were heated for 5 min at 105°C with SDS-PAGE loading buffer [Tris/HCl (200 mM) pH 6.8, glycerol (10% v/v), SDS (2% w/v), Bromophenol Blue (0.1% w/v)]. The samples were separated by SDS-PAGE. Gels were fixed in 10% acetic acid and 10% methanol, dried and exposed to a phosphorimager plate. A FLA-7000 bioimager (Fujifilm) was used to scan the plate to obtain the image.

### Sample preparation for mass spectrometry analysis

Samples were prepared for redox proteomics as previously described (van der Reest et al., 2018) with some modifications. Microsomes were incubated in the presence of the reductive system as described above, either in the presence or absence of Trx1. After 60 min incubation at 30°C, microsomes were lysed in a buffer containing SDS (4%), triethylammonium bicarbonate (50 mM) pH 8.5 and unlabelled iodoacetamide (55 mM; light IAA, ^12^C_2_H_4_INO; Sigma) to alkylate free cysteine thiols. For each experimental replicate, 30 µg of protein was subsequently tagged with iodoacetamide labelled with stable isotope (heavy IAA, ^13^C_2_D_2_H_2_INO; Sigma) to alkylate reversibly oxidised cysteines. Differentially alkylated proteins were precipitated using trichloroacetic acid (TCA) and digested first with endoproteinase Lys-C (enzyme:lysate ratio 1:33) for 1 h, followed by overnight digestion with trypsin (enzyme:lysate ratio 1:33). The digested peptides from each experiment, and a pool sample, were differentially labelled using the Tandem Mass Tag (TMT) 10-plex reagent set (Thermo Scientific). Fully labelled samples were mixed in equal amounts and desalted using 100 mg Sep Pak C18 reverse phase solid-phase extraction cartridges (Waters).

### LC/MS/MS protocol

We analysed three different experimental conditions. For each condition, we analysed three biological replicates, which were labelled with nine tags of the TMT 10-plex. The remaining TMT tag was used to label a pool generated by mixing equal amounts of each sample, which was used to normalise the data using the internal reference scaling method (Plubell et al., 2017).

TMT-labelled peptides were analysed as previously described (van der Reest et al., 2018) with minor modifications. First, peptides were fractionated using high pH reverse phase chromatography on a C18 column [150×2.1 mm internal diameter; Kinetex EVO (5 μm, 100 Å)] on a HPLC system (LC 1260 Infinity II, Agilent). A two-step gradient was applied: from 1-28% buffer B in 42 min, then from 28-46% buffer B in 13 min to obtain a total of 21 fractions for LC/MS/MS analysis.

Fractionated peptides were separated by nanoscale C18 reverse-phase liquid chromatography using an EASY-nLC II 1200 (Thermo Scientific) coupled to an Orbitrap Fusion Lumos mass spectrometer (Thermo Scientific). Elution was carried out using a binary gradient with buffer A (2% acetonitrile) and B (80% acetonitrile), both containing 0.1% formic acid. Samples were loaded with 6 µl of buffer A into a 50 cm fused silica emitter (New Objective) packed in-house with ReproSil-Pur C18-AQ, 1.9 μm resin (Dr Maisch, Ammerbuch, Germany). Packed emitter was kept at 50°C by means of a column oven (Sonation) integrated into the nanoelectrospray ion source (Thermo Scientific). Peptides were eluted at a flow rate of 300 nl/min using different gradients optimised for three sets of fractions: 1-7, 8-15 and 16-21. The initial percentage of buffer B (%B) was kept constant for 3 min, then a two-step gradient was used, all with 113 min for step one and 37 min for step two. The %B was changed as follows: for F1-7, %B was 2 at the start, 17 at step one and 26 at step two. For F8-14, %B was 4 at the start, 23 at step one and 35 at step two. For F15-21, %B was 6 at the start, 27 at step one and 43 at step two. All gradients were followed by a washing step (95% B) of 10 min followed by a 5 min re-equilibration step at the initial %B of each gradient, for a total gradient time of 168 min. Eluting peptides were electrosprayed into the mass spectrometer using a nanoelectrospray ion source (Thermo Scientific). An Active Background Ion Reduction Device (ESI Source Solutions) was used to decrease air contaminant signal level. The Xcalibur software (Thermo Scientific) was used for data acquisition. A full scan over mass range of 350-1400 m/z was acquired at 60,000 resolution at 200 m/z, with a target value of 500,000 ions for a maximum injection time of 20 ms. Higher energy collisional dissociation fragmentation was performed on the 15 most intense ions, selected through an isolation window of 0.8 m/z, for a maximum injection time of 100 ms, or a target value of 100,000 ions. Peptide fragments were analysed in the Orbitrap at 50,000 resolution.

### MS data analysis

The MS Raw data were processed with MaxQuant software (Cox and Mann, 2008) version 1.6.3.3 and searched with Andromeda search engine (Cox et al., 2011), querying SwissProt (UniProt, 2010). First and main searches were performed with precursor mass tolerances of 20 ppm and 4.5 ppm, respectively, and MS/MS tolerance of 20 ppm. The minimum peptide length was set to six amino acids and specificity for trypsin cleavage was required, allowing up to two missed cleavage sites. MaxQuant was set to quantify on ‘Reporter ion MS2’, and TMT 10-plex was chosen as isobaric label. Interference between TMT channels were corrected by MaxQuant using the correction factors provided by the manufacturer. The ‘Filter by PIF’ option was activated and a ‘Reporter ion tolerance’ of 0.003 Da was used. Modification by light [H(3)NOC(2)] and heavy [HNOCx(2)Hx(2)] iodoacetamide on cysteine residues (carbamidomethylation) were specified as variable, as well as methionine oxidation and N-terminal acetylation modifications; no fixed modifications were specified. The peptide, protein and site false discovery rate (FDR) was set to 1%. MaxQuant outputs were analysed with Perseus software version 1.6.2.3 (Cox and Mann, 2008). The MaxQuant output ModSpecPeptide.txt file was used for quantification of cysteine-containing peptide oxidation, whereas the ProteinGroup.txt file was used for protein quantification analysis. Peptides with a cysteine count lower than one were excluded from the analysis, together with Reverse and Potential Contaminant flagged peptides. From the ProteinGroups.txt file, Reverse and Potential Contaminant flagged proteins were removed, as well as protein groups identified with no unique peptides. The TMT corrected intensities of proteins and peptides were normalised to the median of all intensities measured in each replicate. Only cysteine-containing peptides uniquely assigned to one protein group within each replicate experiment, and robustly quantified in three out of three replicate experiments, were normalised to the total protein levels and included in the analysis. To determine significantly regulated cysteine-containing peptides, a Student's *t*-test with a 5% FDR (permutation-based) was applied using normalised reporter ion intensities.

## Supplementary Material

Reviewer comments
