# Supplementary material for: The mammalian cytosolic thioredoxin reductase pathway acts via a membrane protein to reduce ER-localised proteins
Source: J Cell Sci. 2020 Apr 30;133(8):jcs241976. doi: 10.1242/jcs.241976 (PMC7197872; doi:10.1242/jcs.241976)
Supplement: Reviewer comments [file joces-133-241976_review_history.pdf]

The mammalian cytosolic thioredoxin reductase pathway acts via a membrane protein to reduce ER-localised proteins

Xiaofei Cao, Sergio Lilla, Zhenbo Cao, Marie Anne Pringle, Ojore B. V. Oka, Philip J. Robinson, Tomasz Szmaja, Marcel Van Lith, Sara Zanivan and Neil J. Bulleid
DOI: 10.1242/jcs.241976

Editor: David Stephens

Review timeline
Original submission: 20 November 2019
Editorial decision: 9 January 2020
First revision received: 5 March 2020
Accepted: 10 March 2020

Original submission

First decision letter

MS ID#: JOCES/2019/241976

MS TITLE: The Mammalian Cytosolic Thioredoxin Reductase Pathway Acts via a Membrane Protein to Reduce ER-localised Proteins

AUTHORS: Xiaofei Cao, Sergio Lilla, Zhenbo Cao, Marie Anne Pringle, Ojore Oka, Philip Robinson, Tomasz Szmaja, Marcel Van Lith, Sara Zanivan, and Neil Bulleid
ARTICLE TYPE: Research Article

We have now reached a decision on the above manuscript.

To see the reviewers' reports and a copy of this decision letter, please go to: https://submit-jcs.biologists.org and click on the 'Manuscripts with Decisions' queue in the Author Area. (Corresponding author only has access to reviews.)

As you will see, the reviewers gave favourable reports but raised some critical points that will require amendments to your manuscript. I hope that you will be able to carry these out, because I would like to be able to accept your paper.

I agree with their comments that some additional quantitation would support the conclusions as would validating proteins identified by the redox proteomic experiments along with analysis of the change in redox state if feasible. Their other comments should be self-explanatory and are largely related to changes to the text.

Please ensure that you clearly highlight all changes made in the revised manuscript. Please avoid using 'Tracked changes' in Word files as these are lost in PDF conversion.

I should be grateful if you would also provide a point-by-point response detailing how you have dealt with the points raised by the reviewers in the 'Response to Reviewers' box. Please attend to all of the reviewers' comments. If you do not agree with any of their criticisms or suggestions please explain clearly why this is so.

© 2020. Published by The Company of Biologists under the terms of the Creative Commons Attribution License (https://creativecommons.org/licenses/by/4.0/).

Reviewer 1

Advance summary and potential significance to field

Having shown the a cytosolic source is necessary for reducing disulfide bonds in proteins that reside in the ER lumen, in this clear and concise paper Bulleid and coworkers show that the thioredoxin reductase (TxrR) thioredoxin (Trx) is such a source. Moreover, they show that a membrane protein (complex?) is necessary for transferring reducing equivalent from the cytosol to the ER. These results are important and set the stage for the discovery of such e protein, similar to DsbD in bacteria.

Comments for the author

The paper is clearly and concisely written and the data convincing, and is suitable for publication with minor amendments.

This reviewer is left with three curiosities concerning the data shown in this study.

1. If the source of reducing power is cytosolic, what is that maintains part of the sensor and of ERp57 reduced? Shouldn't one predict that both become completely oxidized, as observed after diamide treatment.
Fig 1B and 1C : How come the ox/red ratio in intact cells and microsomes are the same : one would expect the basal ratio to be higher in microsomes without the active cytosolic reducing power. Is perhaps the ratio artificially set at 1? Even so, the difference between UNT and diamide treated samples is almost the same in both intact cells and microsomes which is unexpected.
Accordingly, ERP57 in SP cells is indeed more oxidized than usually seen in intact cells. Then, why did the authors pre-treat cells with diamide (fig 3B) ?

2. How would one interpret the observations that GSH is sufficient to reduce the ER lumen in semipermeable cells?

3. What is the identity of the proteins that become more oxidized in the MS experiments? In this connections, it would be useful to show the selection window that has been used for MS2 analysis. If heavy and light peptides are not properly separated, then TMT cannot be used. It is also not so clear how the quantity of protein in each sample was normalized.

Reviewer 2

Advance summary and potential significance to field

This is a clear, logical manuscript that provides important insight into the mechanism and function of the reductive pathway in eukaryotic oxidative protein folding. New targets have been identified and the paper opens up the field to understanding how the selectivity of the TrxR1 reduction system is achieved.

Comments for the author

The paper is mostly convincing and well reasoned with some caveats as described below:

Fig 1 introduces the enhanced ER-SFGFP-iE reporter – however, the data interpretation partly depends on how “intact” the microsomes are. It would be reassuring to see evidence for the stability of TCEP coupled to agarose beads, to rule out a contribution from released TCEP in the SP cell experiments. Demonstrating that medium pre-incubated with washed TCEP beads contains no free TCEP could be achieved by a simple in vitro assay, for example.

The choice of ERp57 and ADAM10 as readouts (page 7 and onwards) deserves further explanation: is ERp57 expected to play a central role in the Trx reductive pathway or was it simply chosen as a convenient exemplar? Neither ADAM10 nor ERp57 appears in the significantly changing protein dataset identified in Fig 5.

© 2020. Published by The Company of Biologists under the terms of the Creative Commons Attribution License (https://creativecommons.org/licenses/by/4.0/).

Therefore, it is important for the authors to validate a protein identified by the redox proteomic experiments and show the change in redox state in response to the Trx system using the Western blot assay.

Some quantitation of the proportion of reduced and oxidized forms observed in Figs 3 and 4 is required along with the n; and an indication of the number of technical replicates (of the three biological replicates) for the redox proteomics should be stated.

Minor points:

The authors acknowledge that some of the identifications in Fig 5 may result from proteins co-purifying with microsomes – in this regard it may be worth adding a sentence about PBXIP1, which associates with the cytoskeleton in addition to its role as a soluble transcription factor in the nucleus.

In the methods, why was nuclease treatment not included for the ERp57 SP experiments?

There are some minor typographical errors requiring correction e.g. “None AMS-treated” on page 7; and “were expressed in and purified” on page 18; “Oxidised SP cells was incubated” on page 19.

First revision

<u>Author response to reviewers' comments</u>

Reviewer 1:
1. *Why is neither the sensor nor ERp57 fully oxidised in the absence of a cytosolic reductive pathway?* We always find a small fraction of the sensor or ERp57 is reduced following isolation of either microsomes or semi-permeabilised (SP) cells. This reflects the fact that they are not fully oxidised in the intact cell and once the cytosol is removed, they only become slowly oxidised due to the absence of an oxidising activity such as disulfide formation in newly synthesised proteins. We always use SP-cells on the day they are prepared and microsomes are stored at -80 deg C. Prolonged storage does lead to further oxidation but to obtain full oxidation does require the addition of oxidant such as diamide.

Fig 1B and C: Why is the ox/red ratio the same? We do indeed normalise the ratios as indicated on the y-axis (Fig. 1B,C,D,E) as the absolute fluorescent values vary marginally between experiments. This enables a clearer picture of the changes when displayed on a single graph. As explained above the sensor ratio does not change dramatically after removal of the cytosol as we are also removing the oxidation load on the sensor.

Why is ERp57 in SP cells more oxidised – why pre-treat with diamide? The redox state of ERp57 in intact cells tends to vary with cell-type and confluency. We do not show the redox status of ERp57 in intact cells so there is no comparison made in the paper. Because of this variation we decided to pre-treat with diamide to fully oxidise so that we could more clearly evaluate the extent of reduction. Note that we have replaced figure 3A and E for consistency as these were not pretreated with diamide in the original figure.

2. *How would we interpret the previous observations that GSH can reduce the ER in SP-cells?* The reviewer is referring to previous work which showed that GSH when added externally to SP-cells can reduce ER proteins. We refer to this work in our introduction (line 73 -84). Clearly in these circumstances GSH does reduce ER proteins though its role in intact cells and in the rearrangement of non-native disulfides is less clear. We have covered previously our speculations on the role of GSH versus a thioredoxin based systems for reduction both in recent reviews and papers as cited in the introduction.

3. *What is the identity of the proteins that become more oxidised in the MS experiment?*

© 2020. Published by The Company of Biologists under the terms of the Creative Commons Attribution License (https://creativecommons.org/licenses/by/4.0/).

All the proteins whose oxidised thiols become modified (oxidised or reduced) are illustrated in the cartoon in figure 5. To make this clearer we have modified the figure 5 and the table to indicated which proteins become more oxidised or more reduced following the 60 min incubation period.
Provide details of the selection window used for the MS2 analysis and how the quantity of protein in each sample was normalised.
The isolation window for the fragmentation of TMT labelled peptides was set to 0.8 m/z (= 0.4 m/z on each side of the selected precursor). This prevents co-selection of heavy/light iodoacetamide peptide pairs even at charge state higher than five, which is the maximum charge state allowed for MSMS selection in the method. We have modified the methods section accordingly.

Regarding the data normalisation of the redox proteomic experiment, we applied three steps of normalisation on the peptide reporter ion intensity. First, we normalised by the median intensities of each replicate, then every peptide was normalised by the parent protein levels and finally, to be able to compare heavy-light IAA TMT10plex, we normalised using the internal reference scaling method. We have added a paragraph in the methods and a reference to the scaling method. (Plubell DL, Wilmarth PA, Zhao Y, Fenton AM, Minnier J, Reddy AP, Klimek J, Yang X, David LL, Pamir N., Extended Multiplexing of Tandem Mass Tags (TMT) Labeling Reveals Age and High Fat Diet Specific Proteome Changes in Mouse Epididymal Adipose Tissue. Mol Cell Proteomics. 2017 May;16(5):873-890)

Reviewer 2:
1. *Figure 1 introduces the roGFP reporter and the interpretation depends on how intact the microsomes are. An analysis of the presence or absence of free TCEP in medium incubated with TCEP beads would be reassuring.* The experiments comparing the ability of free TCEP to reduce microsomal roGFP indicate a clear difference before and after solubilisation with detergent. The experiment with immobilised TCEP adds to the conclusion that TCEP is acting through a membrane protein to bring about reduction of roGFP. We have repeated the experiment with buffer incubated with TCEP beads and included this as a control in a replacement figure 1E. We have updated the text accordingly. We also carried out a DTNB assay of the wash buffer and did not detect any TCEP (not shown). We feel that the new figure 1E is more convincing as there is a significant effect of the TCEP beads on the redox status of microsomal roGFP which is not seen with the buffer preincubated with the TCEP-beads.
2. *The choice of ERp57 and ADAM10 as readouts deserves further explanation: is ERp57 expected to play a central role in the Trx reductive pathway or was it simply chosen as a convenient exemplar?* ERp57 was chosen for the western blot analysis as we predict it may well be involved in the reductive pathway due to its known interaction with a variety of substrates that form complex disulfide patterns and have the potential to form non-native disulfides. We chose the ADAM10 domain as it was shown to be a substrate of ERp57 and also forms non-native disulfides. The mass proteomics experiment offers much higher resolution of changes to redox status than can be achieved by carrying out AMS treatment and looking for changes by western blotting. However, it does rely upon our ability to detect the modified peptide which is not always the case. The western blot approach also relies upon exogenous expression of tagged proteins due to the sensitivity of the assay. We are fortunate the ERp57 does show a nice shift upon AMS treatment following AMS treatment and so was a convenient way to evaluate the consequence of the addition of a cytosolic reductive pathway on an ER protein. We would argue that the reproducible mass spectrometry data we have is more robust than the AMS /western approach and provides us with a clear demonstration that the redox status of several ER proteins is changed specifically in the presence of the TrxR1 system. Further experiments will need to be now carried out to evaluate the role of the individual proteins we have identified but this is beyond the scope of this paper.
3. *Some quantification of the proportion of reduced and oxidised forms observed in Figs 3 and 4 is required along with the n; and an indication of the number of technical replicates for the redox proteomics should be stated.* We have now indicated the % reduced in each of the ERp57 western blots (Fig 3). Note that we have replaced Figure 3A and 3E with new gel images as we repeated this experiment (x3) as in the original experiments the SP-cells were not pretreated with diamide. All gels in figure 3 are now directly comparable.
The quantification of Fig 4 is not straightforward as all the protein is oxidised – it just forms different disulfide patterns following incubation with the TrxR1 system. Hence the result indicates a change to the disulfide pattern which can be quite clearly discerned qualitatively. We do not carry out technical replicates for our redox proteomics due to the length of runs and costs. We rely

© 2020. Published by The Company of Biologists under the terms of the Creative Commons Attribution License (https://creativecommons.org/licenses/by/4.0/).

upon obtaining consistent results from biological replicates which is more robust than using technical replicates. We have included additional text in the methods section to clarify this point. *Minor points:* We have added a comment about PBXIP1 in the figure legend. We do not need to nuclease treat the SP-cells for the ERp57 experiment we are not carrying out in vitro translation. We nuclease treat for the translations to remove endogenous RNA. If the nuclease treatment or absence of it had any effect that would be controlled for in our experiments. We have corrected the minor typographical errors.

Second decision letter

MS ID#: JOCES/2019/241976

MS TITLE: The Mammalian Cytosolic Thioredoxin Reductase Pathway Acts via a Membrane Protein to Reduce ER-localised Proteins

AUTHORS: Xiaofei Cao, Sergio Lilla, Zhenbo Cao, MARIE ANNE PRINGLE, OJORE OKA, Philip Robinson, Tomasz Szmaja, Marcel Van Lith, Sara Zanivan, and Neil Bulleid
ARTICLE TYPE: Research Article

I am happy to tell you that your manuscript has been accepted for publication in Journal of Cell Science, pending standard ethics checks.

Thank you for your careful revisions which I consider really do strengthen this work. I have concluded that it was not necessary to return this to the reviewers.

© 2020. Published by The Company of Biologists under the terms of the Creative Commons Attribution License (https://creativecommons.org/licenses/by/4.0/).
